# A call for action: Educating pharmacists and pharmacy students in behaviour change techniques

**DOI:** 10.1016/j.rcsop.2023.100287

**Published:** 2023-06-10

**Authors:** Caitlin Liddelow, Barbara A. Mullan, Hayley Breare, Tin Fei Sim, Darren Haywood

**Affiliations:** a School of Psychology, University of Wollongong, Wollongong, New South Wales, 2500, Australia; b enAble Institute, Curtin University, Bentley, Western Australia, 6102, Australia; c Western Australian Cancer Prevention Research Unit, Curtin University, Bentley, Western Australia, 6102, Australia; d School of Population Health, Curtin University, Bentley, Western Australia, 6102, Australia; e School of Pharmacy, Curtin Medical School, Curtin University, Bentley, Western Australia 6102, Australia; f St. Vincent's Hospital Melbourne, Mental Health, Fitzroy, Victoria 3065, Australia; g Turner Institute for Brain and Mental Health and School of Psychological Sciences, Monash University, Clayton, Victoria 3800, Australia

**Keywords:** Health promotion, Community pharmacy, Upskilling, Health professional

## Abstract

The increasing impact of chronic disease, including cancer and heart disease on mortality signifies a need for the upskilling of health professionals in health behaviour change. Solely providing education and information to patients is generally not sufficient to change behaviour, and for any change to be sustained. The nature of pharmaceutical practice allows pharmacists to have frequent contact with patients in the community. Historically, pharmacists have often effectively engaged with patients to assist with behaviour change initiatives related to smoking cessation, weight loss or medication adherence. Unfortunately, such initiatives do not work for everyone, and more tailored and varied interventions are urgently needed to reduce the effects of chronic disease. In addition, with greater inaccessibility to hospitals and GP's (e.g., appointment wait times), it is imperative that pharmacists are upskilled in providing opportunistic health behaviour change techniques and interventions. Pharmacists need to practice to their full scope consistently and confidently, including the use of behavioural interventions. The following commentary therefore describes and provides recommendations for the upskilling of pharmacists and pharmacy students in opportunistic behaviour change. We outline nine key evidence-based behaviour change techniques, the active-ingredients of a behaviour change intervention, that are relevant to common encounters in professional practice by pharmacists, such as improving adherence to medications/treatments and health promotion initiatives. These include social support (practical and emotional), problem solving, anticipated regret, habit formation, behaviour substitution, restructuring the environment, information about others' approval, pros and cons, and monitoring and providing feedback on behaviour. Recommendations are then provided for how this upskilling can be taught to pharmacists and pharmacy students, as well as how they can use these techniques in their everyday practice.

Health professionals globally, including pharmacists, are encouraged to engage in and promote preventative practice with their patients.^1^^,^^2^ With an increase in non-communicable disease (e.g., diabetes, cancer) and illness comorbidity, there is an even greater need for preventative practice from health professionals.^3^ Current training focuses on teaching health professionals the importance of educating patients to change the patients' current behaviour.^4^ However, whilst increasing knowledge is a prerequisite for behaviour change, it is insufficient on its own, and other techniques are required.^5^ Ruppar et al^6^ suggest no intervention with the aim of changing behaviour should solely focus on patient education. Rather, patient education works best when it is combined with more active behavioural approaches, such as behaviour change techniques (BCTs).^7^ BCTs can be described as the ‘active ingredients’ of a behaviour and are often the components that need to change for the target behaviour to change. An example of a common BCT is ‘social support’, either in the form of emotional support (e.g., holding a patient's hand whilst getting a vaccine) or practical support (e.g., a patients' family member driving them to the pharmacy to fill a prescription).^7^ Pharmacists are particularly well-placed to utilise BCTs within their practice and have the potential to reduce the pernicious effects of chronic disease on mortality,^8^ as well as to reduce the demand on hospital emergency departments and General Practice (GP) clinics. There is a need for pharmacists to practice to their full scope, consistently and confidently in line with the modern clinical and patient-centred approach of pharmacy, including the use of behavioural interventions.^9^^,^^10^ It is therefore important to educate pharmacists on BCTs and behavioural interventions to ensure that they are knowledgeable in BCTs and confident to opportunistically facilitate change in their patients' behaviour^.^

Despite public health policies such as the Make Every Contact Count from the National Health Service UK^11^ having included recommendations for preventative practice through applying behaviour change interventions, uptake by health professionals is still inconsistent and sporadic.^11^^,^^12^ We call for urgent attention and consideration to be given to the inclusion of education and training aimed at supporting healthcare professionals to deliver behaviour change interventions and/or BCTs to their patients. Similar calls for action have also been targeted at other health care sectors, such as mental health services.^13^

Currently, pharmacists provide some effective behaviour change initiatives such as smoking cessation and weight management.^14^^,^^15^ Whilst these initiatives are important, they do not work for everyone and the need for more tailored, and varied, behaviour change techniques and interventions^16^ are urgently required. In addition, current pharmacy-based behaviour change interventions can be time and resource-demanding, especially since the COVID-19 pandemic where the roles and responsibilities of pharmacists expanded, meaning interventions may not always be implemented.^17^, ^18^, ^19^ Thus, education in providing short, tailored and *opportunistic* behaviour change techniques is needed to ensure successful prevention and management of chronic diseases in a profession with greater demands. The use of BCTs in pharmacy practice is a simple, efficient (i.e., is not time or resource demanding) and cost-effective way to address prevalent communicable and chronic diseases.^12^^,^^20^

Keyworth and colleagues^1^ identified three methods to improve health professionals' delivery of opportunistic behaviour change, with the primary method being the training of health professionals in BCTs and interventions. Many of the reported challenges of implementing opportunistic behaviour change initiatives in practice, such as health professionals not feeling as though it is their responsibility, being unsure of how to communicate the advice, or being short on time,^21^^,^^22^ can also be targeted and reduced through the introduction of this training. Health professionals have also agreed on the importance of continued training and education of effective health care practice and roles.^23^ Currently, some pharmacy training and additional courses provide introductory education in behaviour change techniques and interventions, particularly that of motivational interviewing.^24^^,^^25^ However, there is contention around the effectiveness of motivational interviewing in primary care settings as an intervention to change health behaviour.^26^ Therefore, more robust and evidence-based behaviour change techniques must be introduced and taught to pharmacy students and practising pharmacists.

A recent pilot study conducted with health professionals involved in the care of patients with heart failure found after a 2-h behaviour change workshop participants were more knowledgeable in BCTs, had greater perceived control in using BCTs and reported greater intention to use them in their practice.^27^ The workshop was also shown to be highly feasible and acceptable in the sample of health professionals. Given the nature of the profession, pharmacists are often the first point of contact, and have frequent contact with patients,^28^ placing them in a strong position for introducing and reinforcing BCTs with patients who may need assistance. The use of simple and effective BCTs by pharmacists will allow for more far-reaching improvements in pharmacy practice, and adherence to medication/treatment guidelines and health provision information. Improvements in adherence to treatments or a greater uptake of health initiatives, can be conducive to positive health outcomes for patients, such as increased quality of life, and decreased risk of comorbidity or mortality.^29^

Below, we describe and provide recommendations for nine BCTs we believe are important for including in the teaching of behaviour change to pharmacists, globally. These BCTs are derived from the Taxonomy of Behaviour Change Techniques^7^ and the Cards for Change tool.^30^ These nine BCTs have been shown to be some of the most successful in improving adherence to treatments and engaging patients in health initiatives across health-settings,^31^ two things which pharmacists are likely to encounter in their professional practice. However, it is important to acknowledge that behaviour, generally, is difficult to change,^5^ and the positive effects of using or recommending BCTs may not always change a patients' behaviour. Nevertheless, delivering opportunistic behaviour change is a brief and easy way to promote behaviour change. It is also important that pharmacists, and those educating them, know that BCTs are often best combined and used in conjunction with each other.^7^ It is also important that pharmacists know that each of the below BCTs can be used in two ways (i) directly implemented by the pharmacist, or (ii) be recommended by a pharmacist to their patient, without direct involvement.

One of the most used BCTs for behaviour change is ‘*social support*’.^7^^,^^32^ It can be provided in the form of practical or emotional support. Practical support can be skills or tools provided by the pharmacist themselves (e.g., providing blister packs/monitored dosage systems to support medication adherence), that the patient can use to ensure they are supported in their target behaviour. This practical support can also be provided by friends and family of the patient (e.g., driving the patient to the pharmacy), if this is more suitable for the patient. Additionally, emotional support can be provided by health professionals, family or friends through words of advice, encouragement, and praise for performance of the intended behaviour.^7^ For example, if a pharmacist knows a patient has difficulties with vaccines, to ensure the patient attends the appointment, a pharmacist may suggest that a family member or friend comes along to the appointment also. The use of social support has previously been found to be effective in a pharmacy-based intervention aimed at improving medication adherence in older adults,^33^ and individuals with adequate health literacy.^34^

One way to ensure a patient reaches their goals is through ‘*problem solving*’. Problem solving is a BCT that involves prompting the patient to identify factors that may be influencing the behaviour and working with them to generate solutions to these barriers.^7^ A patient may express having difficulty taking their medications due to the large number they have been prescribed. The pharmacist or assistant may work directly with the patient to identify the barriers and difficulties with them taking their medications and suggest ways to overcome these barriers. For example, writing a medication schedule on paper to place on the fridge and in the bathroom, so the patient can refer back to the schedule if they are unsure. The use of this BCT can take considerable time if some solutions are unsuccessful, and so is of most benefit for patients who regularly visit the same pharmacist. Solutions developed by the pharmacist or patient should involve planning and establishing specific actions that need to be undertaken to achieve the wanted behaviour. A systematic review by French and colleagues^35^ showed that interventions incorporating problem solving led to significantly increased behaviour compared to interventions that did not include this BCT. More specifically, problem solving has been shown to be effective in improving diabetes self-management and reducing HbA1c levels in both adults and children.^36^

An important factor to consider when changing health-related behaviours is the natural consequences that may occur resulting from the occurrence of a behaviour, or the absence of the positive health-related behaviour. Specifically, the BCT ‘*anticipated regret*’ encourages awareness of the potential negative consequences that may result from the occurrence of the undesired behaviour or lack of engaging in the desired one.^7^ Anticipated regret has been successfully used in colorectal cancer screening interventions to highlight the negative consequences and potential regret of not engaging in or returning the necessary testing kits.^37^ Pharmacists can use this BCT in their practice to highlight the potential regret of not adhering to treatment guidelines or engaging in health initiatives such as preventative screening or vaccination.

‘*Habit formation*’ is one of the only BCTs that encourages maintenance of a behaviour over time.^7^ The development of a habit occurs through consistent rehearsal and repetition of the target behaviour within the same context, in response to the same cue.^38^ This BCT can be useful for patients starting a new behaviour or reducing an old one,^39^ and is often implemented in conjunction with the BCT ‘*restructuring the environment’*. A randomised controlled trial aimed at reducing sedentary behaviour in office workers found that the use of daily automatic reminders to ‘get up and move’ significantly increased habit strength for taking movement breaks.^40^ If a patient discloses to a pharmacist, they need to increase their physical activity (or decrease their sedentary behaviour) to reduce their risk of cardiovascular disease, but are struggling to engage in exercise each day, a pharmacist could recommend and work with the patient to implement similar reminders throughout the day to help them develop a habit of engaging in simple physical activities.

‘*Behaviour substitution*’ is used when there is a need to replace an unwanted behaviour, such as smoking, with a healthier or more positive behaviour to achieve the desired outcome.^7^ Behaviour substitution has been shown to be effective in a randomised controlled trial aimed at reducing sugar-sweetened beverage consumption. The use of a healthier substitution drink (e.g., diet drink or water) was effective in reducing the strength of a habit for consuming drinking sugary drinks.^41^ If a pharmacist is assessing a patient's blood pressure and notices it is high, they may suggest behaviour substitution. For example, a patient may consume large amounts salt in their diet, but a pharmacist could use this BCT and recommend using reduced‑sodium table salt or purchasing low salt products at the supermarket, in attempts to lower their blood pressure.

A BCT associated with the repetition and substitution group of techniques, but is stand-alone, relates to the environmental and contextual cues around the target behaviour. The use of specific cues has been shown to promote the development of habitual behaviours,^42^ and sometimes an environment needs to be ‘restructured’ to assist in this habit formation (i.e., ‘*restructuring the environment’*). This might include adding reminders in the environment, removing physical/social elements that might facilitate/prevent the target behaviour, or adding objects or cues.^7^^,^^42^ Specifically, Stawarz and colleagues^43^ found that contextual cues (e.g., other daily task/behaviours) were better at supporting medication adherence than reminders (e.g., alarms) because even if the routine changes, these cues still exist. A pharmacist may work with their patient, who is struggling with treatment adherence, to devise cues for a necessary behaviour that will work in their specific context.

Comparison of behaviour is a BCT that is known as ‘*information about others’ approval*’ and relates to the awareness of the perceptions of how others may approve/disapprove of the behaviour.^7^ This may include a pharmacist providing factual and/or visual information, as well as anecdotal evidence and opinions from those of importance or interest to the patient. This BCT may be particularly helpful for patients who fear judgement for engaging or disengaging in a behaviour.^7^ Providing information about others' approval has been recommended as one way to promote vaccine uptake, especially in patients who may be hesitant.^44^^,^^45^ For example, pharmacists and pharmacy assistants could inform vaccine hesitant patients of the large percentage of the population who are already vaccinated for the current strain of the flu, which illustrates the approval of the vaccine by many. Similarly, a 2019 review of mass media campaigns by Flowers et al.^46^ found that providing information about others' approval increased HIV testing amongst men who have sex with men. A pharmacist may therefore reiterate the importance of HIV testing from the point of view of a future partner, who would approve of this preventative measure.

In changing health-related behaviours, it is important to make a comparison of outcomes using ‘*pros and cons*’. This BCT entails creating a decisional balance between the pros and cons of engaging or not engaging in behavioural change,^7^^,^^30^ and is likely a BCT that many pharmacists have used before. This technique helps identify areas where support might be needed. For example, a pharmacist may use this technique when trying to assist a patient in smoking cessation and assist in identifying some of the ‘pros’ of quitting smoking (e.g., saving money). A systematic review of interventions aimed at increasing attendance at gyms identified that interventions using ‘pros and cons’ had the largest effect sizes compared to those that did not.^47^

Lastly, feedback and monitoring are valuable to encourage regular performance of a target behaviour. The BCT ‘*monitoring and providing feedback about performance*’ (or lack thereof) is one way to do this. It is particularly helpful to use this technique if the patient is having trouble with a behaviour. Feedback should be offered in a supportive environment that encourages the person to reflect on their behaviour and identify areas where they are seeking feedback.^7^ Additionally asking the patient to monitor their behaviours, whether it is smoking, diet or physical activity can help patients identify areas that impede their progress. For example, Mairs and Mullan^48^ found that monitoring sleep behaviour was effective at improving sleep in college students. If a pharmacist is working with a patient experiencing sleep difficulties, the pharmacist can suggest the patient keeps a sleep diary, or uses an app, to track their sleep. Similarly, if a patient is concerned about their behaviour when under the influence of alcohol (i.e., engaging in unsafe sex), a pharmacist may recommend monitoring and recording their alcohol consumption to reflect on it. The use of this BCT has shown promise in reducing alcohol consumption in several remotely delivered interventions.^49^

In this commentary, we call for the education of pharmacists and pharmacy students in behaviour change techniques. We have highlighted and discussed, with evidence, nine BCTs that can be effectively and easily incorporated into pharmacy education, training, and professional development. These nine BCTs are some of the most successful in improving adherence to treatment regimens and engaging patients in health initiatives.^31^ The combination of an increase in non-communicable diseases, illness comorbidity, and a lack of engagement and confidence in using behaviour change from health professionals, means there is an urgent need for this upskilling.

## Funding

This research received no specific grant from any funding agency in the public, commercial, or not-for-profit sectors.

## Data access statement

Not applicable.

## Declaration of Competing Interest

The authors declare no conflicts of interest.

## Data Availability

Not applicable.

## References

[1] Keyworth C., Epton T., Goldthorpe J., Calam R., Armitage C.J. (2020). Delivering opportunistic behavior change interventions: a systematic review of systematic reviews. Prev Sci.

[2] Toklu H.Z., Hussain A. (2013). The changing face of pharmacy practice and the need for a new model of pharmacy education. J Young Pharm.

[3] Kruk M.E., Nigenda G., Knaul F.M. (2015). Redesigning primary care to tackle the global epidemic of noncommunicable disease. Am J Public Health.

[4] Heath G., Cooke R., Cameron E. (2015). A theory-based approach for developing interventions to change patient behaviours: a medication adherence example from paediatric secondary care. Healthcare..

[5] Arlinghaus K.R., Johnston C.A. (2018). Advocating for behavior change with education. Am J Lifestyle Med.

[6] Ruppar T.M., Dobbels F., Lewek P., Matyjaszczyk M., Siebens K., de Geest S.M. (2015). Systematic review of clinical practice guidelines for the improvement of medication adherence. Int J Behav Med.

[7] Michie S., Wood C., Johnston M. (2013). The behaviour change technique taxonomy (BCTTv1) of 93 hierarchically-clustered techniques: testing reliability of the taxonomy in specifying the content of behaviour change interventions. Ann Behav Med.

[8] Rahayu S.A., Widianto S., Defi I.R., Abdulah R. (2021). Role of pharmacists in the Interprofessional care team for patients with chronic diseases. J Multidiscip Healthc.

[9] Hussein R., Whaley C.R.J., Lin E.C.J., Grindrod K. (2021). Identifying barriers, facilitators and behaviour change techniques to the adoption of the full scope of pharmacy practice among pharmacy professionals: using the theoretical domains framework. Res Soc Adm Pharm.

[10] Tsuyuki R.T., Bond C. (2019). The evolution of pharmacy practice research—part I: time to implement the evidence. Can Pharm J.

[11] Public Health England (2016).

[12] Keyworth C., Epton T., Goldthorpe J., Calam R., Armitage C.J. (2018). Are healthcare professionals delivering opportunistic behaviour change interventions? A multi-professional survey of engagement with public health policy. Implement Sci.

[13] Haywood D., Mullan B.A., Liddelow C., Rossell S., Castle D. (2022). A call for the increased education and use of behaviour change techniques in mental health services. Gen Hosp Psychiatry.

[14] Hijazi M.A., Shatila H., El-Lakany A., al Rifai H, Aboul-Ela M, Naja F. (2020). Role of community pharmacists in weight management: results of a national study in Lebanon. BMC Health Serv Res.

[15] Brown T.J., Todd A., O’Malley C. (2016). Community pharmacy-delivered interventions for public health priorities: a systematic review of interventions for alcohol reduction, smoking cessation and weight management, including meta-analysis for smoking cessation. BMJ Open.

[16] Lustria M.L.A., Noar S.M., Cortese J., Van Stee S.K., Glueckauf R.L., Lee J. (2013). A Meta-analysis of web-delivered tailored health behavior change interventions. J Health Commun.

[17] Bragazzi N., Mansour M., Bonsignore A., Ciliberti R. (2020). The role of hospital and community pharmacists in the management of COVID-19: towards an expanded definition of the roles, responsibilities, and duties of the pharmacist. Pharmacy..

[18] Agomo C.O., Ogunleye J., Portlock J. (2020). Enhancing the public health role of community pharmacists – a qualitative research Utilising the theoretical domains framework. Innov Pharm.

[19] Pantasri T. (2022). Expanded roles of community pharmacists in COVID-19: a scoping literature review. J Am Pharm Assoc.

[20] Laverack G. (2017). The challenge of behaviour change and health promotion. Challenges..

[21] Keyworth C., Epton T., Goldthorpe J., Calam R., Armitage C.J. (2019). ‘It’s difficult, I think it’s complicated’: Health care professionals’ barriers and enablers to providing opportunistic behaviour change interventions during routine medical consultations. Br J Health Psychol.

[22] Vogt K.S., Johnson J., Conner M., Armitage C.J., Keyworth C. (2023). Barriers and enablers to delivering opportunistic behaviour change interventions during the COVID-19 pandemic: a qualitative study in healthcare professionals. Br J Health Psychol.

[23] Newlon J.L., Murphy E.M., Ahmed R., Illingworth K.S. (2023). Determining and regulating scope of practice for health care professionals: a participatory, multiple stakeholder approach. Res Soc Adm Pharm.

[24] Goggin K., Hawes S.M., Duval E.R. (2010). A motivational interviewing course for pharmacy students. Am J Pharm Educ.

[25] Duff A.J.A., Latchford G.J. (2013). Motivational interviewing for adherence problems in cystic fibrosis; evaluation of training healthcare professionals. J Clin Med Res.

[26] Morton K., Beauchamp M., Prothero A. (2015). The effectiveness of motivational interviewing for health behaviour change in primary care settings: a systematic review. Health Psychol Rev.

[27] Mullan B., Liddelow C., Haywood D., Breare H. (2022). Behavior change training for health professionals: evaluation of a 2-hour workshop. JMIR Form Res.

[28] Ilardo M.L., Speciale A. (2020). The community pharmacist: perceived barriers and patient-centered care communication. Int J Environ Res Public Health.

[29] Jimmy B., Jose J. (2011). Patient medication adherence: measures in daily practice. Oman Med J.

[30] Pearson E., Byrne-Davis L., Bull E., Hart J. (2018). Behavior change techniques in health professional training: developing a coding tool. Transl Behav Med.

[31] van Achterberg T., Huisman-de Waal G.G.J., Ketelaar N.A.B.M., Oostendorp R.A., Jacobs J.E., Wollersheim H.C.H. (2011). How to promote healthy behaviours in patients? An overview of evidence for behaviour change techniques. Health Promot Int.

[32] Haywood D., Lawrence B.J., Baughman F.D., Mullan B.A. (2021). A conceptual model of long-term weight loss maintenance: the importance of cognitive, empirical and computational approaches. Int J Environ Res Public Health.

[33] Patton D.E., Ryan C., Hughes C.M. (2020). Development of a complex community pharmacy intervention package using theory-based behaviour change techniques to improve older adults’ medication adherence. BMC Health Serv Res.

[34] Johnson V.R., Jacobson K.L., Gazmararian J.A., Blake S.C. (2010). Does social support help limited-literacy patients with medication adherence?. Patient Educ Couns.

[35] French D.P., Olander E.K., Chisholm A., Mc Sharry J. (2014). Which behaviour change techniques are most effective at increasing older adults’ self-efficacy and physical activity behaviour? A systematic review. Ann Behav Med.

[36] Fitzpatrick S.L., Schumann K.P., Hill-Briggs F. (2013). Problem solving interventions for diabetes self-management and control: a systematic review of the literature. Diabetes Res Clin Pract.

[37] O’Carroll R.E., Chambers J.A., Brownlee L., Libby G., Steele R.J.C. (2015). Anticipated regret to increase uptake of colorectal cancer screening (ARTICS): a randomised controlled trial. Soc Sci Med.

[38] Phillips L.A., Mullan B.A. (2022). Ramifications of behavioural complexity for habit conceptualisation, promotion, and measurement. Health Psychol Rev.

[39] Mergelsberg E.L.P., Mullan B.A., Allom V., Scott A. (2020). An intervention designed to investigate habit formation in a novel health behaviour. Psychol Health.

[40] Luo Y., Lee B., Wohn D.Y., Rebar A.L., Conroy D.E., Choe E.K. (2018). Proceedings of the 2018 CHI conference on human factors in computing systems.

[41] Judah G., Mullan B., Yee M., Johansson L., Allom V., Liddelow C. (2020). A habit-based randomised controlled trial to reduce sugar-sweetened beverage consumption: the impact of the substituted beverage on behaviour and habit strength. Int J Behav Med.

[42] Bartle T., Mullan B., Novoradovskaya E., Allom V., Hasking P. (2019). The role of choice on eating behaviours. Br Food J.

[43] Stawarz K., Rodríguez M.D., Cox A.L., Blandford A. (2016). Understanding the use of contextual cues: design implications for medication adherence technologies that support remembering. Digit Health.

[44] Marshall S., Fleming A., Sahm L.J., Moore A.C. (2023). Identifying intervention strategies to improve HPV vaccine decision-making using behaviour change theory. Vaccine..

[45] Marshall S., Moore A.C., Fleming A., Sahm L.J. (2022). A video-based behavioral intervention associated with improved HPV knowledge and intention to vaccinate. Vaccines (Basel).

[46] Flowers P., Riddell J., Boydell N., Teal G., Coia N., McDaid L. (2019). What are mass media interventions made of? Exploring the active content of interventions designed to increase HIV testing in gay men within a systematic review. Br J Health Psychol.

[47] Rand M., Norman P., Goyder E. (2020). A systematic review of interventions to increase attendance at health and fitness venues: identifying key behaviour change techniques. BMC Public Health.

[48] Mairs L., Mullan B., Self-Monitoring vs. (2015). Implementation intentions: a comparison of behaviour change techniques to improve sleep hygiene and sleep outcomes in students. Int J Behav Med.

[49] Howlett N., García-Iglesias J., Bontoft C. (2022). A systematic review and behaviour change technique analysis of remotely delivered alcohol and/or substance misuse interventions for adults. Drug Alcohol Depend.

